# A Biodegradable, Sustained-Released, Prednisolone Acetate Microfilm Drug Delivery System Effectively Prolongs Corneal Allograft Survival in the Rat Keratoplasty Model

**DOI:** 10.1371/journal.pone.0070419

**Published:** 2013-08-05

**Authors:** Yu-Chi Liu, Yan Peng, Nyein Chan Lwin, Subbu S. Venkatraman, Tina T. Wong, Jodhbir S. Mehta

**Affiliations:** 1 Tissue Engineering and Stem Cell Group, Singapore Eye Research Institute, Singapore, Singapore; 2 Singapore National Eye Centre, Singapore, Singapore; 3 School of Materials Science and Engineering, Nanyang Technological University, Singapore, Singapore; 4 Ocular Therapeutics and Drug Delivery Research Group, Singapore Eye Research Institute, Singapore, Singapore; 5 Department of Clinical Sciences, Duke-NUS Graduate Medical School, Singapore, Singapore; Shanghai Jiao Tong University School of Medicine, China

## Abstract

Frequent and long-term use of topical corticosteroids after corneal transplantation is necessary to prevent graft rejection. However, it relies heavily on patient compliance, and sustained therapeutic drug levels are often not achieved with administration of topical eye drops. A biodegradable drug delivery system with a controlled and sustained drug release may circumvent these limitations. In this study, we investigated the efficacy of a prednisolone acetate (PA)-loaded poly (d,l-lactide-co-ε-caprolactone) (PLC) microfilm drug delivery system on promoting the survival of allogeneic grafts after penetrating keratoplasty (PK) using a rat model. The drug release profiles of the microfilms were characterized (group 1). Subsequently, forty-eight PK were performed in four experimental groups: syngeneic control grafts (group 2), allogeneic control grafts (group 3), allogeneic grafts with subconjunctivally-implanted PA microfilm (group 4), and allogeneic grafts with PA eye drops (group 5; n = 12 in each). PA-loaded microfilm achieved a sustained and steady release at a rate of 0.006–0.009 mg/day, with a consistent aqueous drug concentration of 207–209 ng/ml. The mean survival days was >28 days in group 2, 9.9±0.8 days in group 3, 26.8±2.7 days in group 4, and 26.4±3.4 days in group 5 (*P* = 0.023 and *P* = 0.027 compared with group 3). Statistically significant decrease in CD4+, CD163+, CD 25+, and CD54+ cell infiltration was observed in group 4 and group 5 compared with group 3 (*P*<0.001). There was no significant difference in the mean survival and immunohistochemical analysis between group 4 and group 5. These results showed that sustained PA-loaded microfilm effectively prolongs corneal allograft survival. It is as effective as conventional PA eye drops, providing a promising clinically applicable alternative for patients undergoing corneal transplantation.

## Introduction

Corneal transplantation is the most prevalent transplant procedure worldwide [1]. In the USA, 46196 corneal transplantation were done in 2011 [2]. Although newer forms of selective tissue transplantation, e.g., deep anterior lamellar keratoplasty, reduce the risk of endothelial rejection, penetrating keratoplasty (PK) is still the most common keratoplasty procedure worldwide [3]. Despite the development of immunosuppressants, immunological graft rejection remains the primary cause of graft failure [3], [4]. The majority of rejection episodes occur in the first year with the average period of onset at 8 months after surgery [5], [6]. The risk of graft rejection after PK has been shown to be 10.6% in year 1 and 20% by 5 years [1], [7], [8]. However, in high-risk patients the rejection rate may be greater than 60% during the first year after PK [9], [10]. After a rejection episode, approximately 61% of cases can be treated successfully by medical therapy, whereas approximately 39% of cases will fail to respond and will require a repeat PK [5], [7]. Even in the grafts that survive a rejection episode, there will be a significant reduction in the corneal endothelial cell count. In patients that require a re-graft, the survival rate of the new graft decreases substantially depending on the number of previous grafts [11], [12]. Graft rejection, repeated transplantation, and a growing demand of corneas have lead to a global shortage of corneas available for transplantation [13]. One potential way to mitigate this shortage is to prolong graft survival by improving compliance with medication to reduce the risk of graft rejection and hence failure.

Topical corticosteroids have been established as the gold standard for the prevention and treatment of corneal allograft rejection for over 50 years [14]. Practice preferences amongst UK corneal surgeons reported that 5.5% of surgeons prescribed topical corticosteroid indefinitely to low-risk patients, and 17% of surgeons used it indefinitely for high-risk patients [15]. Prolonged use of topical corticosteroid has been shown to be beneficial for the prevention of graft rejection after PK, and long-term use of low dose corticosteroids has been recommended, even in non-high-risk cases [4]. However, long-term use is highly dependent on patient compliance. The proportion of patients that are non-compliant who are on long-term topical eye drops ranges from 5% to 80% [16]. In addition, topical eye drops often have a short duration of action and low bioavailability, and hence frequent application is required. This may result in discomfort from ocular surface toxicity. Therefore, various attempts have been made to address the limitations related to eye drops.

Several novel drug delivery platforms have been explored to overcome the above challenges and to obtain higher therapeutic efficacy and sustained release, and these include liposomal formulation, microspheres, nanoparticle delivery, and polymeric implants [13], [17]–[21]. Liposomes, microspheres, and nanoparticles have been reported to be good vehicles for the incorporation of immunosuppressants [21], intraocular pressure (IOP)-lowering agents [22], and anti-vascular endothelial growth factor (VEGF) drugs [13] when they were injected subconjunctivally or intravitreally. However, it is challenging to reverse the drug effects from these particle-based delivery systems. When loading corticosteroids onto a drug delivery carrier, one of the concerns is the ability to reverse unwanted corticosteroid related side effects, e.g., elevation of IOP, exacerbation of bacterial and viral infections, and posterior subcapsular cataract formation. Compared to particle delivery systems, removing an implant microfilm from an eye would allow easy reversal of unwanted side effects. Furthermore, a polymeric implant has the capability of loading more drugs in weight, and can be fabricated into various dimensions to modulate the amount and duration of drug release [23]. The use of a biodegradable polymeric implant to deliver a sustained drug level in the eye is therefore an attractive option.

Among different biodegradable microfilm polymers used for drug delivery, poly[d,l-lactide-co-glycolide] (PLGA) copolymers are commonly used [18], [19]. In this study, we used poly [d,l-lactide-co-ε-caprolactone] (PLC) copolymer microfilm. In comparison with PLGA, PLC is more hydrophobic, as the caprolactone ester bonds of the copolymer are not easily hydrolyzed. Because of this slower hydrolysis rate, PLC microfilms degrade more slowly and therefore achieve longer release profiles [24], [25]. Polymeric structural difference in crystallinity also affects degradation rates. PLGA is an amorphous copolymer and hence is more easily degradable than PLC copolymer, which has semi-crystalline structure. Our previous studies have confirmed that PLC microfilms degrade slower than PLGA microfilms both *in vitro* and *in vivo* [20]. Moreover, PLGA copolymers have a higher glass transition temperature than PLC copolymers, which makes PLGA copolymers physically hard, while PLC copolymers are soft and elastic [26], [27]. Hence we chose the softer material for the implant fabrication as it minimizes the possibility of surgical trauma during the implantation procedure as well as of extrusion after implantation. We have previously demonstrated the safety/efficacy and biocompatibility of the prednisolone acetate (PA)-loaded PLC microfilm in reducing postoperative inflammation and prolong bleb survival in a rabbit glaucoma filtration surgery model [20], [28]. We have also shown that this subconjunctivally-implanted drug delivery system provided therapeutically effective levels of PA in the anterior chamber in a small animal model [29], indicating it may also be suitable to be used following corneal transplantation. In this study, we aimed to evaluate the efficacy of the biodegradable, sustained-released, PA-loaded PLC drug delivery system on corneal graft survival using a rat allogeneic penetrating keratoplasty model.

## Materials and Methods

### Drug Delivery System

Details on the fabrication of drug delivery system have been described in our published articles [20], [29]. Briefly, polymeric microfilms were prepared using a solution casting method [30]. Copolymer PLC (d,l-lactide to ε-caprolactone molar ratio was 70/30, with intrinsic viscosity of 1.6 dl/g; Purac Far East, Singapore) and prednisolone 21-acetate (≥97%) (Sigma-Aldrich, Singapore) with a predetermined drug loading percentage of 40wt% were dissolved in dichloromethane to form a polymer solution. This drug-polymer mixture was cast on a glass plate using an automatic film applicator. Subsequently, the films were dried under a fume hood for one day, followed by drying in a vacuum oven at 37°C until the solvent level was less than 1% of the total weight, as measured using a thermo-gravimetric analyzer (TGA, TA instruments Q500). After drying, the microfilms were manually cut into the standard size of 3.5×4.5×0.1 mm.

All the samples were sterilized by ethylene oxide (ETO) at 37°C in Tan Tock Seng Hospital (Singapore) prior to implantation.

### Animals

A total of 72 female Lewis rats (Rtl-l^vl^) and 18 female Fisher rats (Rtl-l^1vl^) aged 8- to 10-week-old were used. All animals were treated in accordance with the tenets of the Association for Research in Vision and Ophthalmology Statement for the Use of Animals in Ophthalmic and Vision Research, and the protocol was approved by the Institutional Animal Care and Use Committee of SingHealth (IACUC SingHealth approval number 2012/SHS/699). All surgical procedures were performed under general anesthesia with intraperitoneal injection of ketamine hydrochloride (50–75 mg/kg) and xylazil (5–8 mg/kg).

The animals were divided to five groups:

Group 1: Thirty-six eyes from 18 Lewis rats were used for assessment of the drug release of the PA-loaded microfilms and PA eye drops.

Group 2: Twelve corneal grafts from Lewis rats were transplanted onto the right eye of another twelve Lewis rats (syngeneic control group).

Group 3: Twelve corneal grafts from Fisher rats were transplanted onto the right eye of twelve Lewis rats (allogeneic control group).

Group 4: Twelve corneal grafts from Fisher rats were transplanted onto the right eye of twelve Lewis rats. The recipient eyes also underwent subconjunctival PA-loaded microfilms implantation (PA microfilm group).

Group 5: Twelve corneal grafts from Fisher rats were transplanted onto the right eye of twelve Lewis rats. The recipient eyes were treated with prednisolone acetate eye drops three times daily (Pred Forte**®**, Allergan; PA eye drop group).

### Microfilm Drug Release Study

Rats in group 1 were used for assessment of drug release profile from the microfilms. After the animals had been adequately anaesthetized, 18 eyes were implanted with the PA microfilms. A 4.5 mm superior-temporal fornix based subconjunctival pocket was created via blunt dissection and the microfilm was inserted. Closure with two 10-0 sutures (nylon, Ethicon) was performed to ensure secure placement of the microfilm. At 2, 4 and 12 weeks after insertion, the microfilms were retrieved (n = 3 for each time point). The retrieved microfilms were rinsed with deionized water then dried in a vacuum oven at 37°C over a week. The dried samples were dissolved in 10 mL of acetonitrile, and the amount of residual drug in each microfilm was determined by high-performance liquid chromatography (HPLC). The amount of drug released was quantified by calculating the difference in the initial loaded drug and the residual drug. The percentage of the cumulative drug release was also derived accordingly. In addition, the PA level in the aqueous humor was also measured. At 2, 4 and 12 weeks, aqueous humor was aspirated using 30-gauge needles from the rats with implanted PA microfilm (n = 3 for each time point) or with PA eye drops treatment three times daily (n = 3 for each time point). PA concentrations were then determined using HPLC.

### Penetrating Keratoplasty

Rats were anesthetized and orthotropic corneal transplantation was performed. Mydriasis in the eyes of both the donors and recipients was done by local application of 1% mydriacyl**®** (Alcon), and then the donors were euthanized with overdose intraperitoneal pentobarbitone (60–150 mg/kg). Corneal grafts were obtained with a diameter of 3.5 mm, using the previously described “underwater technique” [31]. After the recipient corneas were removed with a 3.0 mm trephine, the grafts were transplanted onto the recipients with eight 10-0 interrupted sutures (nylon, Ethicon). The anterior chamber of the eye was reformed by injection of balanced salt solution (BSS). For the PA microfilm group, the microfilms were inserted to the subconjunctival space as described above. At the end of the operation, a tarsorrhaphy was performed with two 7-0 interrupted sutures (silk, Ethicon). Topical tobramycin ointment (Alcon) was given 4 times daily for the initial 4 days. The graft sutures were removed 2 weeks after operation. The eyes complicated by cataract, infection, or hyphema were excluded.

### Clinical Evaluation

After surgery, all the recipient eyes were observed by slit lamp biomicroscopy and anterior segment optical coherence tomography (ASOCT; RTVue; Optovue, Inc, Fremont, CA) every other day until day 28. The grafts were evaluated using a previously established scoring system [32]. The scoring system assessed graft opacity (0–4), edema (0–4) and neovascularization (0–4) (Table 1). A graft was considered rejected when the combined score (rejection index, 0–12) was equal to or exceeded 6 [32]. ASOCT was used to assess the graft contour, integrity and position, as well as to obtain measurements of the central thickness of the grafts.

**Table 1:** Clinical scoring scheme for the severity of corneal graft rejection.

Type/Score	Clinical finding
Graft opacity	
0	No opacity
1	Slight opacity, details of iris clearly visible
**Type/Score**	**Clinical finding**
Graft opacity	
0	No opacity
1	Slight opacity, details of iris clearly visible
2	Some details of it is no longer visible
3	Pronounced opacity, pupil still recognizable
4	Total opacity
Graft edema	
0	No edema
1	Mild edema
2	Pronounced edema with raised transplant
3	Pronounced edema with small bleb
4	Pronounced edema with large bleb
Graft neovascularization	
0	No vessels
1	Vessels appearing in the corneal bed
2	Vessels appearing in the graft periphery
3	Vessels extending deeper
4	Vessels extending to the center

For the PA microfilm group, the Hackett-McDonald ocular scoring system [33] was also used to evaluate conjunctival congestion (0–3), swelling (0–4) and discharge (0–3) around the microfilm insertion sites weekly.

### Histopathological and Immunohistochemistry Analyses

Two rats from each transplantation group (group 2–5) were euthanized with overdose intraperitoneal pentobarbitone (60–150 mg/kg) at 14 days after PK. The remaining ten rats from each transplantation group were observed for 28 days for evaluation of graft survival. The eyeballs were embedded in OCT cryo-compound (Leica Microsystems, Nussloch, Germany), and then were cut into 7 µm slices using a cryostat (Microm HM 550; Microm, Walldorf, Germany). These sections were subjected to haematoxylin and eosin (H&E) and immunohistochemistry staining. Monoclonal mouse anti-rat antibodies to T-helper cells (CD4, MCA153R), cytotoxic T cells (CD8, MCA48R), interleukin 2 receptor (IL-2R) (CD25, MCA273R), intercellular adhesion molecule 1 (ICAM-1) (CD54, MCA773), macrophages (CD163, MCA342R), and dendritic cells (CD11c, MCA1441) were used. All the antibodies were purchased from Serotec, Oxford, UK. The immune-expression was quantified by counting the number of cells from both the central cornea and graft-host junction in ten non-overlapping sections in each corneal specimen. The cells were counted in a ×100 microscopic field and by a single, masked observer (Y.C.L).

### Statistical Analysis

Time to rejection was analyzed by the Kaplan-Meier method for graft survival analysis and was compared by the log-rank test. All data was expressed as mean ± standard error (SE) unless it was indicated that standard deviation (SD) was used. Statistical comparisons between groups were performed using ANOVA test with Bonferonni correction. All data analyses were done with SPSS software package (SPSS Inc.; Chicago, IL). A *P*<0.05 was considered as significant.

## Results

### Microfilm Drug Release Profile

The PA microfilms demonstrated a steady and sustained daily release of approximately 0.006–0.009 mg/day over 12 weeks and were almost exhausted of drug at 12 weeks. The PA microfilms released 13.1±0.4%, 27.8±2.2% and 99.1±0.1% of the loaded drug at 2, 4 and 12 weeks, respectively.

PA concentrations were detected in the aqueous humor, with levels of 209.1±26.9 and 207.6±23.2 (mean±SD) ng/ml in rats with implanted PA-microfilm at 2 and 4 weeks, and 297.1±61.2 and 266.2±59.7 (mean±SD) ng/ml in rats receiving PA eye drops three times daily at 2 and 4 weeks (group 1). The PA levels for the PA eye drop group were higher than those for the PA microfilm group at 2 and 4 weeks, but they were not statistically significant (*P* = 0.110 and *P* = 0.102, Table 2). The PA concentrations in the aqueous humor in either PA microfilm or PA eye drop group were consistent during the follow-up period of 4 weeks.

**Table 2:** Prednisolone-acetate (PA) concentration in the aqueous humor.

Treatment group	Concentration at 2 week (n = 3; ng/ml)	Concentration at 4 week (n = 3; ng/ml)
Untreated	0	0
40% PA microfilm	209.1±26.9	207.6±23.2
1% PA eye drops	297.1±61.2	266.2±59.7
*P* value*	0.110	0.102

* P value of the comparison between 40% PA microfilm and 1% PA eye drops groups; non-parametric test. Data were expressed as mean ± standard deviation.

### Clinical Evaluation

**Figure 1:**
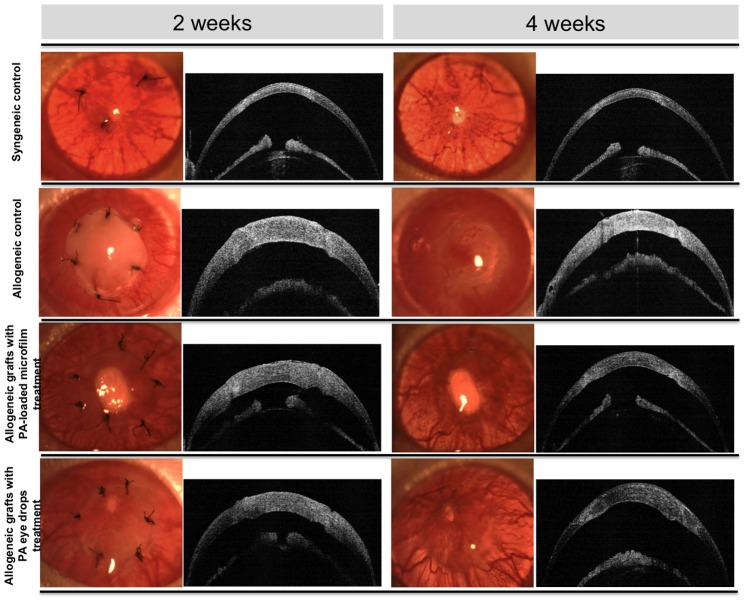
Clinical evaluation of corneal grafts by slit lamp biomicroscopy and ASOCT at 2 and 4 weeks. At 2 weeks, all allogeneic control grafts exhibited rejection episodes with severe graft edema and opacity, whereas grafts from the PA microfilm and PA eye drop groups had minimal graft edema and opacity. At 4 weeks, all allogeneic control grafts progressed to complete opacification and pronounced edema with neovascularization. The grafts from the PA microfilm and PA eye drop groups appeared clear with visible pupillary margin. All syngeneic control grafts remained clear during the follow-up period. On ASOCT, all grafted corneas exhibited good anatomic position without graft-host dehiscence or anterior chamber collapse.

**Figure 2:**
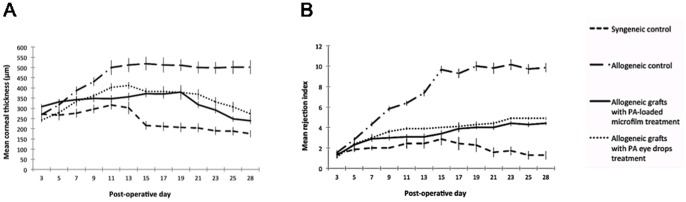
Changes of graft thickness and mean rejection scores with time for different groups. **(**A) The mean central graft thickness measured by ASOCT per time point for different groups. (B) The mean of the graft RI per time point for different groups.

At 2 weeks after surgery, all allogeneic control grafts exhibited rejection episodes with severe graft edema and opacity, whereas grafts from the PA microfilm and PA eye drop groups had minimal graft edema and opacity. All the allogeneic control grafts were rejected by 2 weeks. At 4 weeks after surgery, all allogeneic control grafts progressed to complete opacification and pronounced edema with neovascularization extending from graft-host junction centrally. In contrast, only 2 grafts from the PA microfilm group and two from the PA eye drop group underwent graft rejection clinically on day 24 and day 20 (microfilm group), and on day 19 and day 21 (eye drops group). The remaining 8 grafts from the PA microfilm and PA eye drop groups each appeared clear with visible pupillary margin. All syngeneic control grafts remained clear during the total follow-up time of 28 days. On ASOCT, all grafted corneas exhibited good anatomic position without graft-host dehiscence or anterior chamber collapse (Figure 1). The mean change in central corneal thickness measured by ASOCT against time, in different groups are shown in Figure 2A. All grafts showed an early increase in corneal thickness during the first 2 weeks after PK, and thereafter the thickness declined gradually in the syngeneic control, PA microfilm and PA eye drop groups. The mean corneal thickness in the syngeneic control group declined to a normal rat corneal thickness range at a level of 175.6±17.5 µm at 4 weeks, whereas the allogeneic control grafts were persistently thick, 501.7±34.2 µm at 4 weeks. The mean corneal thickness was 275.0±32.28 µm and 308.7±39.6 µm for the grafts from the PA microfilm group and PA eye drop group respectively at 4 weeks. The grafts treated with PA microfilms or PA eye drop had significantly less mean corneal thickness as compared to the allogeneic control grafts from day 15 onwards (*P* = 0.002 and *P* = 0.014). There was no significant difference between the PA microfilm and PA eye drop groups. The allogeneic PA microfilm group and PA eye drop group both had significantly thicker grafts than the syngeneic control group at day 15–21 (all *P*<0.01 for both groups).

Analysis of the changes of the mean rejection index (RI) revealed that the RI for the allograft control group was significantly higher than those for the PA microfilm and PA eye drop groups from day 7 onwards until day 28 (*P*<0.05 at all time points; Figure 2B). The RI for the syngeneic control and allogeneic control grafts were 2.9±0.6 and 9.6±0.4 at 2 weeks, and 1.3±0.4 and 9.9±0.4 at 4 weeks, whereas the RI for the grafts treated with PA microfilms were 3.4±0.9 at 2 weeks (*P*<0.001 compared with the allogeneic control grafts) and 4.4±0.2 at 4 weeks (*P*<0.001 compared with the syngeneic control or allogeneic control grafts), and the RI for the grafts treated with PA eye drops were 4.0±0.0 at 2 weeks (*P*<0.001 compared with the allogeneic control grafts) and 4.9±0.1 at 4 weeks (*P*<0.001 compared with the syngeneic control and allogeneic control grafts). The changes of the mean scores of graft opacity, edema, and neovascularization with time for different groups are shown in Figure S1A–C.

**Figure 3:**
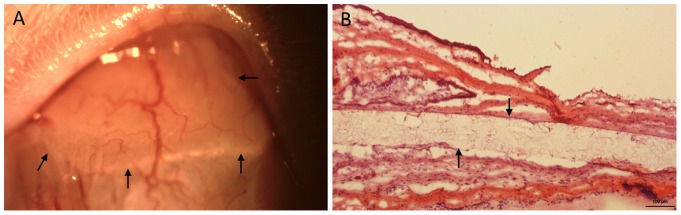
Clinical and histological evaluation of PA microfilm implantation. **(**A) Slit lamp photo showing the subconjunctivally-implanted microfilm at 4 weeks. Arrows indicated the implanted microfilm. (B) Histological section with H&E staining at 4 weeks. Arrows indicated the implanted microfilm. Original magnification×100. Scale bar: 100 µm.

The microfilm insertion sites were also evaluated (group 4). Slit lamp examination revealed mild degree of conjunctival vessels congestion around the insertion site at day 1 after insertion, but it rapidly resolved within 3 days. The mean total Hackett-McDonald ocular scores (0–10) assessing conjunctival congestion, swelling and discharge were very low with a score of 0.11±0.06, 0.04±0.04, 0 and 0 at 1, 2, 3 and 4 weeks respectively. This indicated that the microfilms elicited very minimal inflammation at the insertion sites. The microfilms were placed securely in the subconjunctival space without any evidence of protrusion or dislocation during the study period. There were no clinical signs of infection, neovascularization, bleeding or scarring at the insertion sites (Figure 3A).

**Figure 4:**
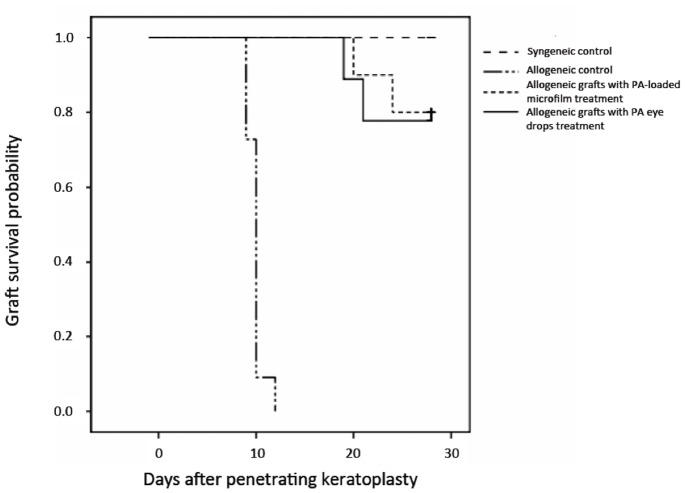
Kaplan-Meier analysis of the rejection-free graft survival. The survival probability at 28 days was 100%, 0%, 80.0% and 76.7% in the syngeneic control, allogeneic control, PA microfilm, and PA eye drop groups, respectively. The PA microfilm and PA eye drop groups had significantly longer survivals as compared to the allogeneic control group (*P* = 0.012 and *P* = 0.014, log-rank test). There was no significant difference between the PA microfilm and PA eye drop groups (*P* = 0.58, log-rank test).

The rejection-free graft survival for all groups are shown in Figure 4 and Table 3. The survival days (mean±SD) was >28 days in the syngeneic control group, 9.9±0.8 days in the allogeneic control group, 26.8±2.7 days in the PA microfilm group (*P* = 0.023 compared with the allogeneic control group), and 26.4±3.4 days in the PA eye drop group (*P* = 0.027 compared with the allogeneic control group). There was no significant difference between the PA microfilm and PA eye drop groups (*P* = 0.67) (Table 3). The survival probability at 28 days was 100% in the syngeneic control group, 0% in the allogeneic control group, 80.0±12.7% in the PA microfilm group (*P* = 0.012 compared with the allogeneic control group, log-rank test), and 76.7±13.4% in the PA eye drop group (*P* = 0.014 compared with the allogeneic control group, log-rank test). There was no significant difference between the PA microfilm and PA eye drop groups (*P* = 0.58, log-rank test).

**Table 3:** Survival time of rat grafts in different groups within study period of 28 days.

Group	Number	Survival days (Mean± SD)	*P* *
Syngeneic control	10	>28	0.018
Allogeneic control	10	9.9±0.8	–
Allogeneic grafts treated with PA microfilm	10	26.8±2.7	0.023
Allogeneic grafts treated with PA eye drops	10	26.4±3.4	0.027

* P value by comparing the allogeneic control group with other groups.

### Histopathological Analysis

**Figure 5:**
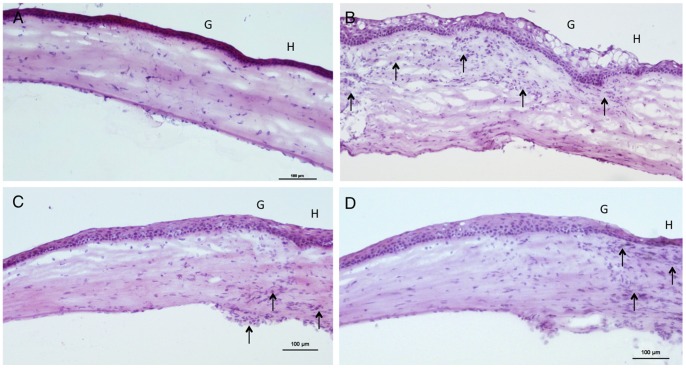
Histological sections with H&E staining for different groups. **(**A) Syngeneic control group. The graft presented normal architecture and thickness with very scant cell infiltrate in the stroma. (B) Allogeneic control group. There was marked graft edema and diffuse infiltration of mononuclear cells in the stroma (arrow). (C) Allogeneic grafts treated with PA microfilms (D) Allogeneic grafts treated with PA eye drops. Thickening of stroma and cellular infiltration (arrow) were appreciably less in the grafts treated with PA microfilms or PA eye drops. G: Graft. H: Host. Original magnification×100. Scale bar: 100 µm.

The syngeneic control grafts had normal architecture with normal corneal thickness and scant infiltrating cells (Figure 5A). The allogeneic control grafts showed marked stromal edema and intense infiltration by mononuclear cells. The infiltration was most marked in the subepithelial and stroma layers and was most evident at the graft-host junction (Figure 5B). Stromal edema and cellular infiltration were notably less in grafts treated with PA microfilms or PA eye drops (Figure 5C,D).

For the rats with PA microfilm insertion, there were minimal inflammatory cells surrounding the implanted microfilms without evidence of excessive scarring or obvious conjunctival atrophy in the subconjunctival space. (Figure 3B).

### Immunohistochemistry Analysis

**Figure 6:**
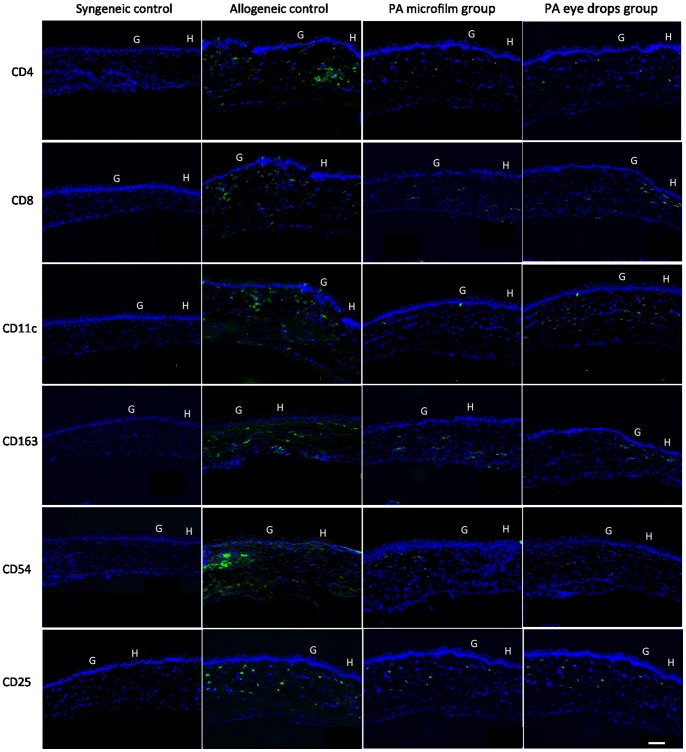
Immunohistochemistry staining for CD4+, CD8+, CD11c+, CD163+, CD54+, and CD25+ cells at graft-host junction for different groups. The grafts treated with the PA microfilms or PA eye drops had less dense and diffuse cell infiltration in comparison with the allogeneic control grafts. The syngeneic grafts were nearly free of immunologic cell infiltration. G: Graft. H: Host. Original magnification ×100. Scale bar: 100 µm.

**Figure 7:**
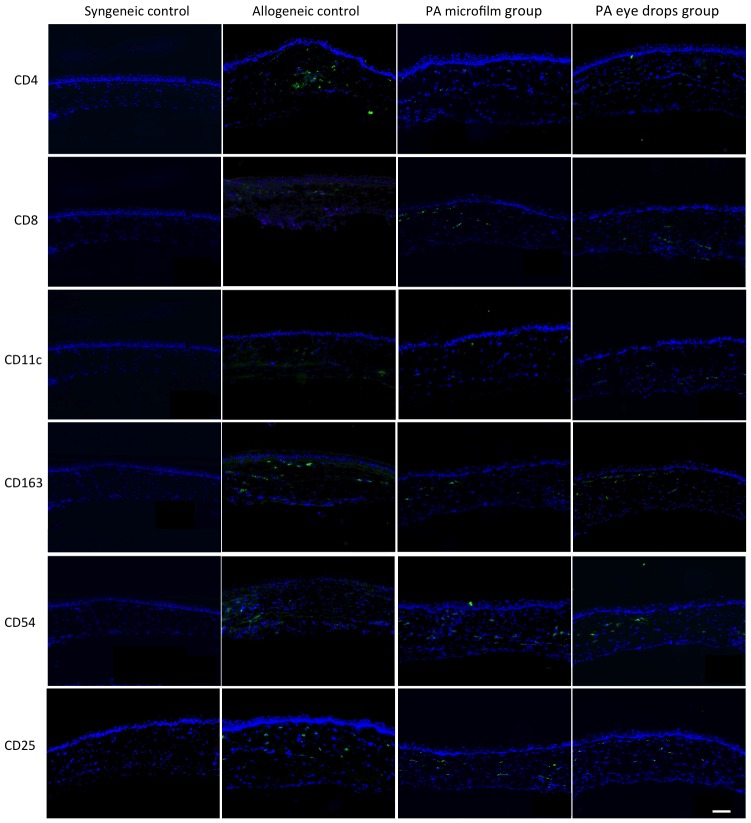
Immunohistochemistry staining for CD4+, CD8+, CD11c+, CD163+, CD54+, and CD25+ cells at central grafts for different groups. The syngeneic grafts were nearly free of immunologic cell infiltration. The grafts treated with the PA microfilms or PA eye drops had less dense and diffuse cell infiltration in comparison with the allogeneic control grafts. G: Graft. H: Host. Original magnification ×100. Scale bar: 100 µm.

**Figure 8:**
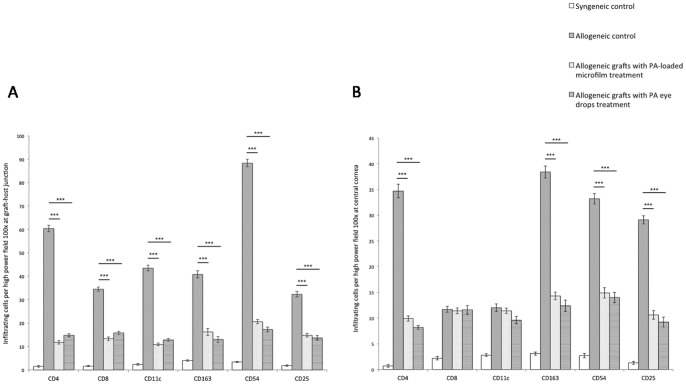
Quantification of CD4+, CD8+, CD11c+, CD163+, CD54+, and CD25+ positively stained cells for different groups. (A) Quantification of positively stained cells at graft-host junction. The grafts from the PA microfilm and PA eye drop groups showed a statistically significantly decreased infiltration of CD4+, CD8+, CD25+, CD54+, CD11c, and CD163+ cells compared to the allogeneic control grafts. (B) Quantification of positively stained cells at central grafts. The grafts from the PA microfilm and PA eye drop groups showed a statistically significantly decreased infiltration of CD4+, CD25+, CD54+, and CD163+ cells compared to the allogeneic control grafts. Error bars represent SE and asterisks indicate *P*<0.001.

The grafts treated with the PA microfilms or PA eye drops had less dense and diffuse cell infiltration for CD4+, CD8+, CD25+, CD54+, CD11c+, and CD163+ cells at either the graft-host junction or central grafts, in comparison with the allogeneic control grafts. The syngeneic grafts had minimal cell infiltration (Figure 6 and Figure 7). After quantification, the grafts from the PA microfilm and PA eye drop groups showed a statistically significantly decreased infiltration of CD4+, CD25+, CD54+, and CD163+ cells compared to the allogeneic control grafts at both the graft-host junction and central grafts (Figure 8, *P*<0.001). There was no statistically significant difference in the amount of CD4+, CD8+, CD25+, CD54+, CD11c+, and CD163+ infiltrating cells between the PA microfilm and PA eye drop groups at both the graft-host junction (*P = *0.28, 0.25, 0.76, 0.56, 0.78 and 0.45, respectively) and central grafts (*P* = 0.67, 0.82, 0.90, 0.82, 0.89, and 0.79, respectively).

## Discussion

In the present study, we have demonstrated that transplants with concomitant PA microfilm implantation produced a statistically significantly prolonged rejection free graft survival compared to allogeneic controls. We have also demonstrated that the PLC sustained PA-loaded microfilm drug delivery system is as effective as conventional PA eye drops, in delaying graft rejection and reducing the immune response after allogeneic corneal transplantation in a rat model.

Currently there are two corticosteroid-loaded drug delivery systems approved by Food and Drug Adminstration (FDA): Retisert® (Bausch & Lomb/pSivida Ltd., Rochester, NY Rochester, NY) and Ozurdex ® (Allergan, Inc., Irvine, CA) [34]. Retisert®, the first approved intravitreal drug implant, is composed of silicon/polyvinyl alcohol (PVA) and is a nonbiodegradable reservoir-type device containing 0.59 mg fluocinolone acetonide. It delivers a low dose of fluocinolone acetonide for a period of approximately 3 years [34], [35]. Ozurdex®, a biodegradable intravitreal implant, uses poly[d,l-lactide-co-glycolide] (PLGA) copolymers as the carrier. It is loaded with 0.7 mg dexamethasone and achieves 6 months of release [34], [36]. However, both of these FDA-approved vehicles are intravitreal implants and are indicated for posterior segment diseases. With respect to anterior segment drug delivery systems, several studies have demonstrated the efficacy of intra-ocular biodegradable implants for the suppression of immune graft rejection in animal models by incorporating cyclosporine [37], rapamycin [38], FK506 [39], or dexamethasone [40] onto drug delivery reservoirs. However, previous studies used drug delivery devices composed of poly[glycolide-co-lactide-co-caprolactone] (PLGC) copolymers and were placed in the anterior chamber. Our drug delivery system used in this study is made of PLC copolymers and can be implanted subconjunctivally, i.e., extra-ocularly. Hence it avoids complications associated with intra-ocular implantation [41].

PLC is a relatively new copolymer that is made of poly(l-lactide) and poly(caprolactone), each of which has been approved by FDA as implantable products. PLC is biodegradable and metabolizes into lactic acid and caproic acid. These monomers are nontoxic and eliminated safely via the krebs cycle by conversion to carbon dioxide and water without causing any foreign-body reactions [34]. In addition to our reports, its use has also been reported in neurological, orthopedic, and cardiovascular research [42]–[44]. Subconjunctival corticosteroids delivery has been shown to provide higher sustained concentrations in aqueous as compared with topical corticosteroids due to the high permeability of sclera relative to cornea [45]–[47]. Implantation of the microfilm in the subconjunctival space can bypass many barriers to topical drug delivery [47]; it is also a simpler and less invasive procedure than anterior chamber implantation and avoids anterior segment complications, e.g., implant migration or peripheral anterior synechiae [41].

The administration of topical corticosteroids at an intensively frequent dosing regime to reduce the postoperative inflammatory response and subsequently suppress the potential rejection reaction remains essential for all types of corneal transplantation procedures [48]. We have shown that our PA microfilm was well tolerated and released steady levels of drug in a sustained manner. This characteristic of the microfilm not only eliminates patients’ dependency but also extends its clinical applications such as following cataract or glaucoma filtration surgery.

Among the clinically available corticosteroid preparations, 1% PA ophthalmic suspension (Pred Forte®) is widely used following corneal transplantation and has excellent ocular penetration [49]. We chose the acetate analogue of prednisolone since it is more hydrophobic, hence it has a greater tendency to remain encapsulated inside PLC microfilms rather than be released quickly. We have previously shown the drug release profile of a PA microfilm is proportional to the size of the microfilm [29]. Hence the latter can be optimized based on the target dose needed in a certain clinical scenario. In the present study, 40% PA 3.5×4.5 mm microfilm (the maximum size possible in a rat eye), was capable of releasing 7.0 ug/day for the first 2 weeks and 6.2 ug/day for the following two weeks. The bioavailability of topically applied eye drops has been predicted to range from only 1% to 5% for lipophilic molecules, such as corticosteroids, due to anatomical and physiological barriers and washout by tears [50]. In a rat eye, one drop of 1% PA ophthalmic solution, which is approximately 8 µl (exceeds the capacity of the rat conjunctival sac and simulates the usual situation when eye drops are given to patients), contains approximately 2.4 µg of PA. In order to avoid bias on analyzing graft survival resulting from a mismatch in the doses between the eye drop and microfilm groups, we decided to apply 1% PA eye drops thrice daily in the experiment (7.2 µg of PA), which provides a daily drug amount close to that of the PA microfilm. Our results showed that both 1% PA eye drops and PA microfilms effectively prevented allograft rejection. However, the 2-week and 4-week cumulative drug amounts were slightly higher in the PA eye drop group (7.2 µg/day for 4 weeks) compared with the PA microfilm group (7.0 ug/day for 2 weeks and 6.2 ug/day for the subsequent 2 weeks). This explains why the aqueous PA concentrations in the PA eye drop group were slightly higher than those in the PA microfilm group (Table 2). However, the PA microfilm still achieved comparable aqueous PA levels to those provided by PA eye drops, with a constant concentration of 207∼209 ng/ml approximately. McGhee et al. [49] reported the concentration after applying 1-drop of 1% PA eye drops in patients undergoing routine cataract surgery was 669.9 ng/ml at 2 hours, reducing to 99.5 ng/ml at 12 hours and 28.4 ng/ml at 24 hours. Comparatively, the PA microfilm provided a steady release at a therapeutic concentration for 4 weeks.

The size of the microfilm in the present study was limited due to the small eyeball of the rat; larger microfilms with greater concentration/weight of loaded drug will be designed for the future human clinical trials. The PA concentration was not detected in the aqueous samples at 12 weeks by HPLC. We postulate this is because the microfilms were almost exhausted of drug at 12 weeks (99.1% of release), leading to the relatively scant amount of aqueous PA.

Corneal graft rejection is a complex immune process mediated by CD4+ T cell [51]. Immunological graft rejection reaction begins at graft-host junction and proceeds centrally [52], hence we observed more cell infiltration at the graft margin. We found a significant reduction of CD4+, CD25+ (IL-2 R), CD54+ (ICAM-1), and CD163+ (macrophage) cells at both the graft-host junction and central graft after treatment with PA microfilms or PA eye drops as compared with non-treatment allogeneic control. Besides inhibiting the chemotaxis and phagocytosis of macrophages [5], corticosteroids block the release of IL-1, IL-3, IL-6 and IL-8 from antigen-presenting cells, subsequently inhibiting IL-2 release, and in turn suppress T cell activity [53]. The expression of ICAM-1, which is thought to play a crucial role in immunological rejection after corneal transplantation [54], was also decreased in the PA microfilm and PA eye drop group. CD8+ T lymphocytes, although present in rejecting grafts, have been reported to be not required to precipitate graft rejection, and CD8-deficient mice reject corneal grafts in a similar manner to fully immune-competent mice [55]. This may explain why there was no statistically significant reduction of CD8+ cells in the PA microfilm and PA eye drop groups as compared with the allogeneic control group. There was no statistically significant difference in CD11c+ cells in all groups.

The rat keratoplasty model is a well-established technique to study immunopathology associated with corneal transplantation [32], [37], [51], [52]. In the allogeneic control grafts, the average survival days of 9.9 days was similar to other previously published studies, in which the survivals ranged from 5.2 to 11.3 days [32], [37], [51]. Unlike some studies in which graft rejection was assessed by simply grading the opacity level alone, we evaluated the rejection by observing the graft opacity, edema and neovascularization collectively and separately [32]. Pan et al. reported the mean RI for the allogeneic control group increased to a score of 10 approximately at day 21 and slightly declined thereafter [32]. Similarly, the mean RI for our allograft control group reached a score of 9.6 at 2 weeks and stayed at a plateau until day 28. Treatments with PA microfilm or PA eye drops significantly reduced the RI and prolonged the graft survival similarly in both groups. In addition, we also demonstrated for the first time, the use of ASOCT imaging to serially measure and compare the graft thickness in a rat PK model.

In conclusion, our PA-loaded PLC microfilm drug delivery system is a new, safe and effective means of drug administration to prevent graft immune rejection and prolong graft survival in the rat penetrating keratoplasty model. It provides a promising alternative to conventional eye drops after corneal transplantation surgery. The biodegradable microfilm has the capacity to be customized to deliver different release profiles depending on different clinical scenarios. Furthermore, as corticosteroids have been used in a broad spectrum of ophthalmic inflammatory conditions, the PA microfilm could also play a role in the treatment of many anterior segment inflammatory disorders.

## Supporting Information

Figure S1 **The mean of the graft opacity, edema and neovascularization scores per time point for different groups.** (A) The mean opacity scores for the allograft control group were significantly higher than those for the PA microfilm and PA eye drop groups from day 7 onwards (*P*<0.001). There was no significant difference in the mean opacity scores between the PA microfilm and PA eye drop groups at all time points, and both groups had higher mean opacity scores than the syngeneic control from day 19 onwards (*P*<0.001). (B) The mean edema scores for the allograft control group were significantly higher than those for the PA microfilm and PA eye drop groups from day 9 onwards (*P*<0.001). There was no significant difference in the mean opacity scores between the PA microfilm and PA eye drop groups at all time points, and both groups had higher mean edema scores than the syngeneic control from day 19 onwards (*P*<0.001). (C) The mean neovascularization scores for the allograft control group were significantly higher than those for the PA microfilm and PA eye drop groups from day 13 onwards (*P*<0.001). There was no significant difference in the neovascularization scores between any groups of the PA microfilm, PA eye drop, and syngeneic groups at all time points.
(TIF)
