# Supplementary figures and images for: A Biodegradable, Sustained-Released, Prednisolone Acetate Microfilm Drug Delivery System Effectively Prolongs Corneal Allograft Survival in the Rat Keratoplasty Model
Source: PLoS One. 2013 Aug 5;8(8):e70419. doi: 10.1371/journal.pone.0070419 (PMC3734265; doi:10.1371/journal.pone.0070419)

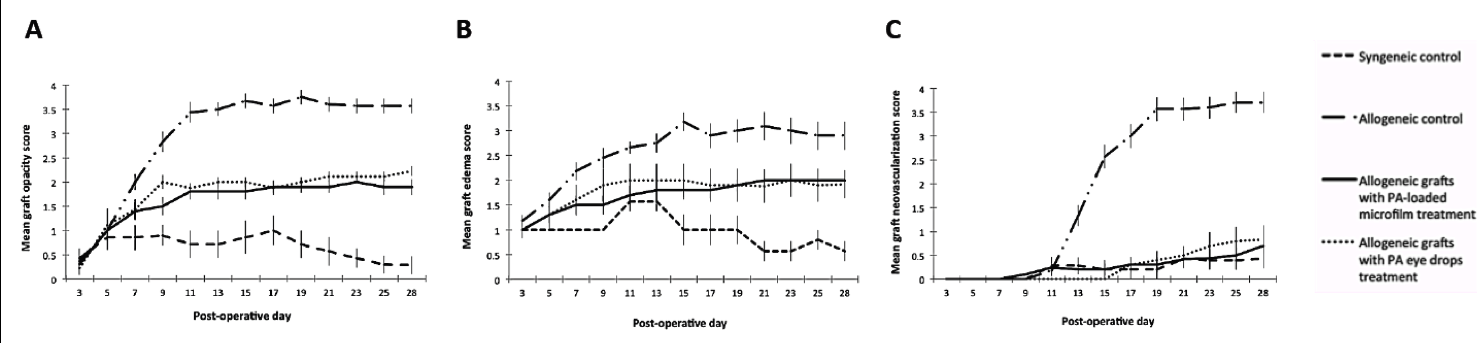

Supplement: Figure S1 — The mean of the graft opacity, edema and neovascularization scores per time point for different groups. (A) The mean opacity scores for the allograft control group were significantly higher than those for the PA microfilm and PA eye drop groups from day 7 onwards (P<0.001). There was no significant difference in the mean opacity scores between the PA microfilm and PA eye drop groups at all time points, and both groups had higher mean opacity scores than the syngeneic control from day 19 onwards (P<0.001). (B) The mean edema scores for the allograft control group were significantly higher than those for the PA microfilm and PA eye drop groups from day 9 onwards (P<0.001). There was no significant difference in the mean opacity scores between the PA microfilm and PA eye drop groups at all time points, and both groups had higher mean edema scores than the syngeneic control from day 19 onwards (P<0.001). (C) The mean neovascularization scores for the allograft control group were significantly higher than those for the PA microfilm and PA eye drop groups from day 13 onwards (P<0.001). There was no significant difference in the neovascularization scores between any groups of the PA microfilm, PA eye drop, and syngeneic groups at all time points. (TIF) [file pone.0070419.s001.tif]
